# Mineralocorticoid receptor deficiency improves the therapeutic effects of mesenchymal stem cells for myocardial infarction via enhanced cell survival

**DOI:** 10.1111/jcmm.14026

**Published:** 2018-12-13

**Authors:** Xinxing Xie, Yunli Shen, Jing Chen, Zheyong Huang, Junbo Ge

**Affiliations:** ^1^ Department of Cardiology Rizhao Heart Hospital Shandong China; ^2^ Department of Cardiology Shanghai East Hospital Tongji University Shanghai China; ^3^ Shanghai Institute of Cardiovascular Disease Zhongshan Hospital Fudan University Shanghai People's Republic of China

**Keywords:** cell survival, mineralocorticoid receptor, myocardial infarction, stem cells

## Abstract

The poor survival of stem cells seriously limits their therapeutic efficacy for myocardial infarction (MI). Mineralocorticoid receptor (MR) activation plays an important role in the pathogenesis of multiple cardiovascular diseases. Here, we examined whether MR silencing in bone marrow derived mesenchymal stem cells (MSCs) could improve MSCs’ survival and enhance their cardioprotective effects in MI. MSCs from male Sprague‐Dawley rats were transfected with adenoviral small interfering RNA to silence MR (siRNA‐MR). MR silencing decreased hypoxia‐induced MSCs’ apoptosis, as demonstrated by Annexin V/7‐AAD staining. The mechanisms contributing to the beneficial effects of MR depletion were associated with inhibiting intracellular reactive oxygen species production and increased Bcl‐2/Bax ratio. In vivo study, 1 × 10^6^ of MSCs with or without siRNA‐MR were injected into rat hearts immediately after MI. Depletion of MR could improve the MSCs’ survival significantly in infarcted myocardium, associated with more cardiac function improvement and smaller infarct size. Capillary density were also significantly higher in siRNA group with increased expression of vascular endothelial growth factor. Our study demonstrated that silencing MR promoted MSCs’ survival and repair efficacy in ischaemic hearts. MR might be a potential target for enhancing the efficacy of cell therapy in ischaemic heart disease.

## 1 INTRODUCTION

Despite the dramatic advances in therapeutic interventions for acute myocardial infarction (AMI), The occurrence of heart failure (HF) after AMI remains the main cause of morbidity and mortality worldwide.^1^, ^2^ As a result of a limited regenerative ability, massive and irreversible loss of cardiomyocytes, followed by the progressive ventricular remodelling is the key problem of HF complicating AMI.^3^ Unfortunately, there are still no fundamental methods to solve this issue. In recent decades, stem cells‐based therapeutic strategies are considered as a promising alternative for the treatment of ischaemic HF. Among several cell types examined in preclinical studies, bone‐marrow derived mesenchymal stem cells (MSCs) seemed to be a preferred cell source for cardiac repair because of their low immunogenicity, the ease of isolation and expansion ex vivo.^4^ Cumulative clinical evidence have proven the efficacy and safety of MSCs therapy in AMI,^5^, ^6^, ^7^, ^8^ and its improvement in cardiac function is comparable with the results from reperfusion and pharmacological therapy.^9^ Recent clinical trials observed a direct relationship between dose and efficacy in cell therapy for ischaemic heart disease.^10^, ^11^, ^12^ However, most transplanted cells died because of the hostile microenvironment after infarction.^13^ Considering a “ceiling effect” of cell therapy for heart disease,^14^ it was a more attractive strategy to promote the MSCs’ survival when compared with unlimited increase in MSCs’ dose to enhance the therapeutic efficacy of stem cell therapy.

The mineralocorticoid receptor (MR), a ligand‐dependent transcription factor, belongs to the nuclear receptor family. MR activation played an important role in the pathogenesis of multiple cardiovascular diseases.^15^, ^16^ Pharmacological MR antagonists showed a beneficial effect in patients with heart failure.^15^, ^17^ As two important hormone ligands, aldosterone and corticosterone plasma levels were elevated after infarction, MR antagonists or MR deletion could suppress cardiomyocytes apoptosis and prevent adverse cardiac remodelling after AMI.^18^, ^19^ In addition, MR could be activated by hypoxia and involved in the pathogenesis of pulmonary hypertension.^20^ Previous studies also revealed that MR activation by aldosterone could impair the function and decrease the number of endothelia progenitor cells (EPCs).^21^ Thus, we hypothesized that MR might be involved in the process of MSCs’ apoptosis after transplantation in AMI, and this study was conducted to verify this hypothesis.

## 2 MATERIALS AND METHODS

### 2.1 Isolation and culturing of rat MSCs

MSCs were isolated from the femur of 100‐120 g male Sprague‐Dawley rats (aged 4‐5 weeks) and expanded as previously described.^22^ The mesenchymal population was isolated based on its ability to adhere to the culture plate. Cultures (low‐glucose Dulbecco's modified Eagle's medium containing 10% foetal bovine serum) were changed every 3‐4 days. Once cultures became 80% confluent, the cells were passaged and plated out at 1:2 to 1:3 dilutions. All cells in our study were 3rd‐4th passage. Animal experiments were approved by the Animal Care and Use Committee of Fudan University in compliance with the “Guide for the Care and Use of Laboratory Animals” published by the National Academy Press (NIH Publication No. 85‐23, revised 1996).

To evaluate MSCs surface antigens, MSCs at passage 4 were incubated with mouse monoclonal anti‐rat CD34, CD44, CD45, CD29, and CD90 (Cyagen). Fluorescence was analysed by flow cytometry.

### 2.2 Adeno‐associated virus (AAV)

AAV‐MR‐siRNA or control vector AAV‐siRNA (AAV‐NC‐siRNA) were synthesized by GenePharma (Shanghai, China). The siRNA sequence for MR is 5′‐CCAACAAGGAAGCCTGAGC‐3′ (position in gene sequence: nucleotide 842‐860), which has been verified previously.^23^ AAV‐NC‐siRNA does not match any known human or rat coding cDNA. All two viral vectors contained enhanced green fluorescent protein (GFP). The AAV‐MR‐siRNA titre was approximately 10^8^ TU/mL.

### 2.3 Cell transfection

MSCs were seeded into 6‐well plates at a density of 1 × 10^6^ cells/mL. After 24 hours of culture, the medium was removed, replaced with serum‐free DMEM containing 10 μL (multiplicity of infection, MOI = 10) of AAV‐MR‐siRNA (siRNA group) or AAV‐NC‐siRNA (NC group) for a further 24 hours. After transfection with siRNA for 48 hours. Transfection efficiency was determined by fluorescence microscopy and fluorescence‐activated cell sorter (FACS) analysis. Alterations of MR mRNA was corroborated by Quantitative Real‐time PCR (qRT‐PCR).

### 2.4 qRT‐PCR for MR expression

Isolation of total RNA from the different treatment groups of MSCs, and their subsequent first strand cDNA synthesis was performed using RNeasy mini kit (QIAGEN, Hilden, Germany) and Omniscript Reverse Transcription kit (QIAGEN) according to the manufacturer's instructions. Primers for amplification of MR and β‐actin are listed below.

MR: sense primer, 5′‐GGT CAA CCC ATT TCC ATT CA‐3′, antisense prime, 5′‐AGG TGG TCC TAA GAT TCC TGA TA‐3′; β‐actin: sense primer, 5′‐CGT AAA GAC CTC TAT GCC AAC A‐3′; antisense primer, 5′‐GGA GGA GCA ATG ATC TTG ATC T‐3′. The detailed method has been described previously.^24^

### 2.5 Cell viability assay

MSCs viability was determined by the Cell Counting Kit‐8 (CCK‐8) assay (Beyotime, Beijing, China) according to the manufacturer's instructions. Briefly, MSCs were seeded into a 96‐well plate. 10 μL of CCK‐8 solution was added to each well and incubated for 2 hours at 37°C. The plate was read at a wavelength of 490 nm.

### 2.6 In vitro model of MSCs injury

MSCs injury was induced under hypoxia for 6 hours in serum‐ and glucose‐free DMEM, which had been described previously,^25^ the hypoxia duration was confirmed from our preliminary experiments. Briefly, cells were washed with PBS and placed in serum‐ and glucose‐free DMEM and then incubated in a sealed, hypoxic Anaerobic Rectangular Jar fitted with a catalyst (BioMe′rieux, Marcy l'Etoile, France, http://www.biomerieux.com) to consume free oxygen. Oxygen tension in the medium, measured by a blood gas analyser, was 33.5 mm Hg within 0.5 hours after being transferred into the hypoxic chamber and maintained at about 22‐24 mm Hg over the experiment time.

### 2.7 Apoptosis assessment

MSCs apoptosis was detected using the fluorescent dye PE Annexin V Apoptosis Detection Kit I (BD PharmingenTM, Franklin Lakes, NJ, USA, http://www.bd.com/) according to the manufacturer's instructions. Briefly, cells were rinsed with ice‐cold PBS and then resuspended in 200 μL of binding buffer. Five microlitres of Annexin V‐PE and 5 μL of 7‐AAD were added to the cells and incubated for 15 minutes at 25°C in the dark. Cells were analysed by FACS within 1‐hour. Ten thousands events were acquired on a FACSC‐LSR (Becton‐Dickinson, San Jose, CA, USA) and analysed with CellQuest (Becton‐Dickinson) software.

### 2.8 Measurement of intracellular reactive oxygen species (ROS)

The production of intracellular ROS was measured using an oxidation‐sensitive fluorescent dihydroethidium (DHE) probe (Vigorous, Beijing, China). The assay was based on the principle that DHE could cross cell membranes and be rapidly oxidized in the presence of ROS, which resulted in the formation of a highly fluorescent form of oxidative ethidium. MSCs were incubated with 10 μmol L^−1^ DHE for 15 minutes at 37°C in the dark, and then washed with PBS. The fluorescence level, as an indicator of ROS production, was detected using fluorescence microscopy. The excitation and emission filters were 488 nm and 590 nm, respectively.

### 2.9 Cell extract preparation and Western Blot analysis

For analysis of protein levels, cells were lysed in lysis buffer and proteins were separated by SDS‐PAGE, followed by blocking in PBS/Tween‐20 containing 2% BSA. The blots were then incubated overnight at 4°C with antibodies specific to Bcl‐2, Bax, vascular endothelial growth factor (VEGF) (Bioworld Technology, Inc., St. Louis Park, MN, USA). These membranes were incubated for 2‐hour with HRP‐ conjugated secondary antibodies. After washing in PBST‐buffer, bands were visualized by SuperSignal West Femto and exposed to radiography film (Bio Rad, Hercules, CA, USA). GAPDH or beta tubulin was used as a loading control.

### 2.10 In vivo studies

#### 2.10.1 Rat AMI model and MSCs transplantation

AMI model was developed in Sprague‐Dawley rats (200‐250 g, aged 6‐8 weeks), as previously described.^26^ The male MSCs were used for the following two‐part experiments as previously described^27^: (a) Gender‐mismatched transplantation for quantifying survival MSCs by real‐time PCR (n = 3/group) and fluorescent microscopy (n = 3/group): 1.0 × 10^6^ of MSCs transfected with AAV‐NC‐siRNA (NC group) or AAV‐MR‐siRNA (siRNA group) in 100 μL of volume were intramyocardially injected at four sites into female recipient hearts; (b) Gender‐matched transplantation for cardiac function comparison: 1.0 × 10^6^ of MSCs transfected with AAV‐NC‐siRNA (NC group) or AAV‐MR‐siRNA (siRNA group) or PBS (PBS group) in 100 μL of volume were intramyocardially injected at four sites into the anterior and lateral aspects of the viable myocardium bordering the infarction of male Sprague‐Dawley rats hearts immediately after AMI with a 30‐gauge needle (n = 5/group). The chest cavity was closed and rats were allowed to recover under a warm light.

#### 2.10.2 Assessment of cardiac function

Left ventricular (LV) function were assessed by transthoracic echocardiography, which was performed at 4 weeks after MI using a 17.5‐MHz phased‐array transducer (Vevo 770, Visualsonics, Toronto, Canada). After the induction of light general anaesthesia, hearts were imaged two dimensionally in long‐axis view at the level of the greatest LV diameter. This view was used to position the M‐mode cursor perpendicular to the LV anterior and posterior walls. The LV end‐diastolic diameters and LV end‐systolic diameters were measured from M‐mode recordings. LV ejection fraction (LVEF) was calculated as follows: LVEF (%) = [left ventricular end‐diastolic dimension (LVID,d)^3^‐left ventricular end‐systolic dimension (LVID,s)^3^]/(LVID,d)^3^ × 100. The percentage of LV fractional shortening (LVFS) was calculated as follows: LVFS (%) = [LVID,d‐LVID,s]/LVID,d×100. All echocardiographic measurements were averaged from at least five separate cardiac cycles.

#### 2.10.3 Measurement of infarct size

Fixed hearts were embedded in paraffin and LV cross‐sections were cut into 5‐μm slices and processed. Myocardial infarct size was determined from three mid‐LV sections after staining with Masson's Staining. The infarct size was defined as the sum of the epicardial and endocardial infarct circumference divided by the sum of the total LV epicardial and endocardial circumferences using computer‐based planimetry. In addition, bright field microscopy was performed on Masson stained histological section to identify islands of myofibres in LV infarcted tissue.

#### 2.10.4 Real‐time PCR for Sry‐gene

Myocardial specimens from different groups were frozen in liquid nitrogen and powdered. The genomic DNA purification was performed using Genomic DNA Isolation Kit (Tiangen, Beijing, China) and the concentration of the purified DNA was determined by spectrophotometry. Real‐time PCR was performed using Takara SYBR^®^ Premix Ex Taq™ in a Bio‐Rad iQ5 optical module. Primers for amplification of rat Y‐chromosome Sry and β‐actin are listed below.

Sry: sense primer, 5′‐GAG GCA CAA GTT GGC TCA ACA‐3′; antisense primer, 5′‐CTC CTG CAA AAA GGG CCT TT‐3′; β‐actin: sense primer, 5′‐CCA TTG AAC ACG GCA TTG‐3′; antisense primer, 5′‐TAC GAC CAG AGG CAT ACA‐3′. The detailed method has been described previously.^26^ The relative PCR products were calculated with the 2^−ΔΔCt^ method, and the results were normalized against β‐actin expression.

#### 2.10.5 Tracking of the GFP^+^ cells injected in infarcted hearts

Quantification of GFP^+^ MSCs was performed on serial sections as described previously.^28^ Rat hearts were harvested at 4 days after MI.^26^, ^29^ Myocardial specimens were frozen in liquid nitrogen and cut from the base (above the ligation) towards the apex until the ligation suture was reached. Beginning at this point, 10 μm sections were collected throughout the entire lesion. After costaining with DAPI, the sections were visualized and photographed in the infarct border zone under microscope. The number of GFP^+^ MSCs were calculated under fluorescent microscopy in randomly selected five high‐power fields (HPF, 200×) in three sections per animal and averaged.

#### 2.10.6 Determination of capillary density

For capillary density analysis, Optimum cutting temperature compound (O.C.T. compound) embedded hearts were sectioned into 5 μm slices. Adjacent sections (taken at the midpoint between LAD ligation site and apex) were stained with antibodies against rat CD31 (Abcam Biochemicals, Cambridge, UK). Capillary density was defined as CD31^+^ endothelial cells per high‐power field (200×). The capillaries were examined in randomly selected five high‐power fields in three sections per animal and averaged.

### 2.11 Statistical analysis

Experiments were repeated at least three times. All data were described as mean ± SEM. To analysis the data statistically, we performed one‐way ANOVA with post hoc analysis and considered a value of *P* < 0.05 as statistically significant.

## 3 RESULTS

### 3.1 Adenoviral vector transfection of MSCs

**Figure 1:**
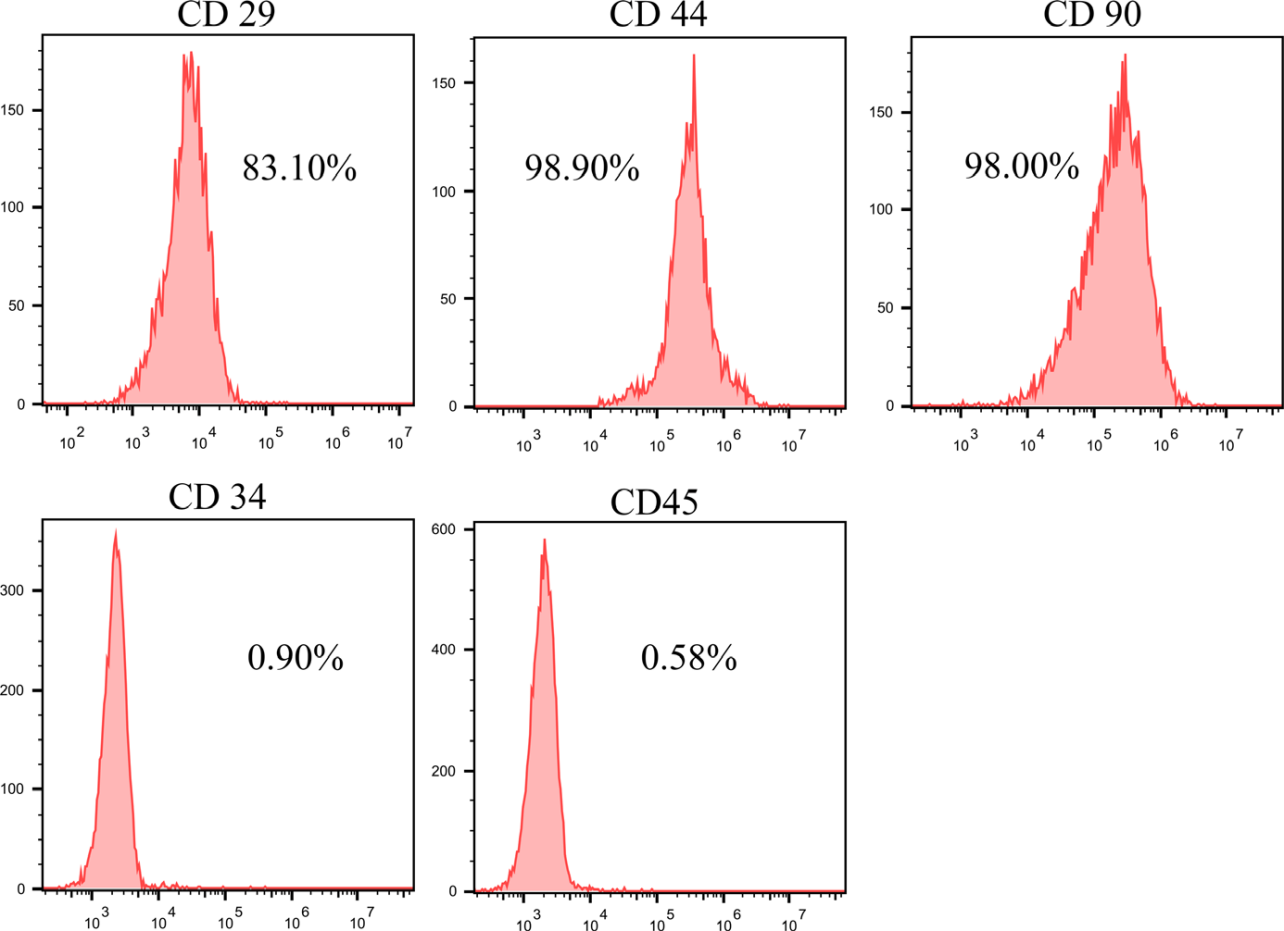
Flow cytometric analysis of surface antigen expression on MSCs. The rat MSCs were positive for CD29,CD44, and CD90 but negative for myeloid/hematopoietic cell‐specific antigens of CD34 and CD45

**Figure 2:**
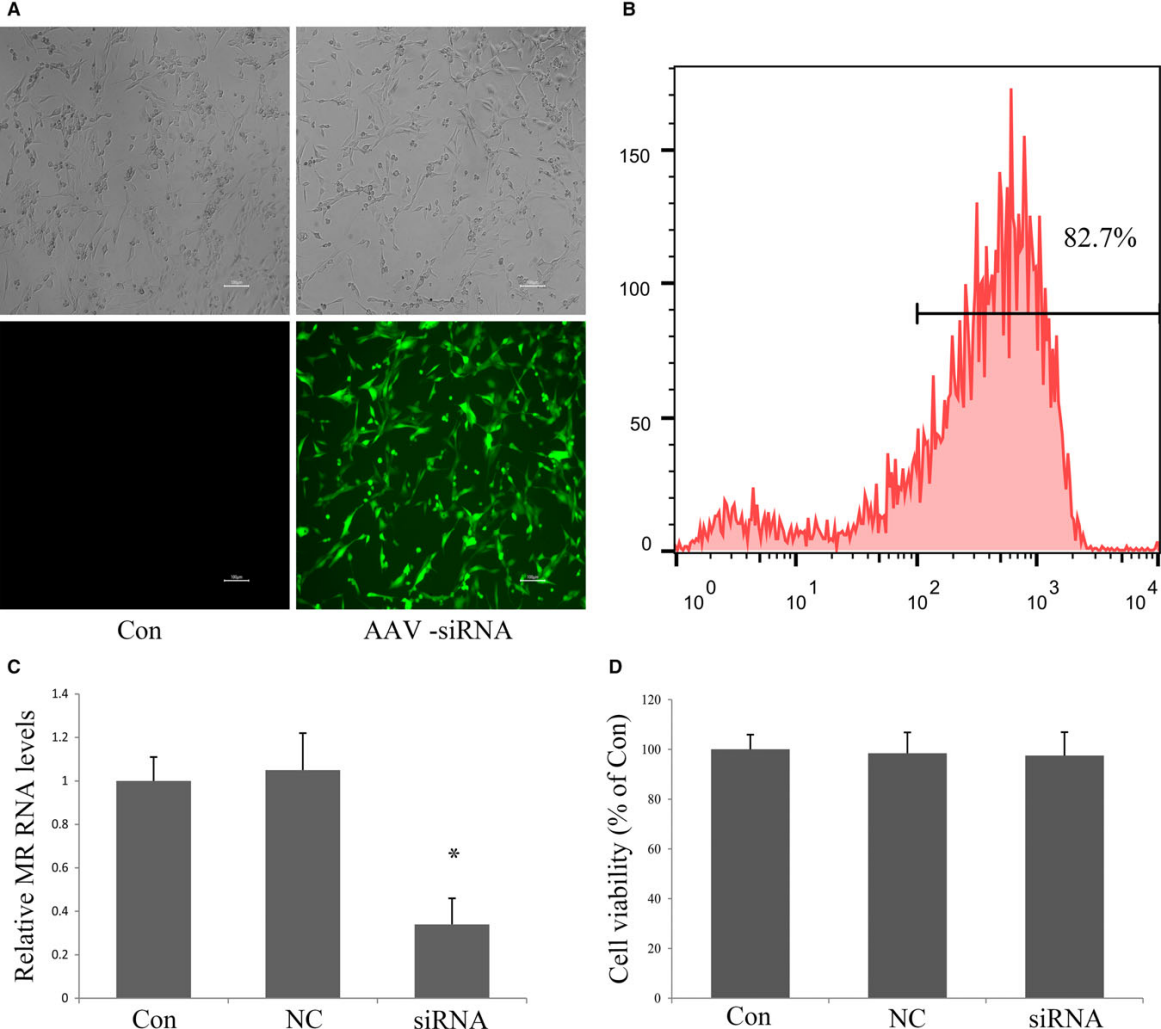
Transfection of MSCs with AAV vector. (A) Representative photomicrograph of MSCs transfected with AAV‐NC‐siRNA/GFP, scale bar: 100 μm. (B) FACS analysis showing the transfection efficiency of GFP transfected MSCs. (C) qRT‐PCR analysis of MR mRNA expression in MSCs 48 h after transfection with AAV‐MR‐siRNA/GFP. (D) AAV‐siRNA/GFP transfection did not affect cell viability examined with CCK‐8 assay. All data were collected from three independent experiments. *P < 0.05 vs control and NC

The MSCs displayed a homogenous spindle‐shaped population and maintained a similar morphology during the subsequent passages. Characterization by flow cytometric analysis showed that most MSCs expressed CD29, CD44 and CD90, but not the hematopoietic markers of CD34 and CD45 (Figure 1). A representative GFP expression was shown in Figure 2. We observed more than 90% of MSCs were transfected by adenoviral vector having 10 MOI (Figure 2A). The transduction efficiency, as determined by the percentage of GFP positive cells was 82.7% based on FACS analysis (Figure 2B). To evaluate the expression of MR mRNA in the genetically modified MSCs in vitro, qRT‐PCR was performed on cell samples at 48 hours after transfection. The results showed that the relative expression of MR mRNA in the siRNA group was significantly lower than those in MSCs and NC group (Figure 2C). Cell viability was comparable among three groups (Figure 2D).

### 3.2 Depletion of MR protected MSCs against apoptosis in vitro and enhanced the survival in infarcted heart

**Figure 3:**
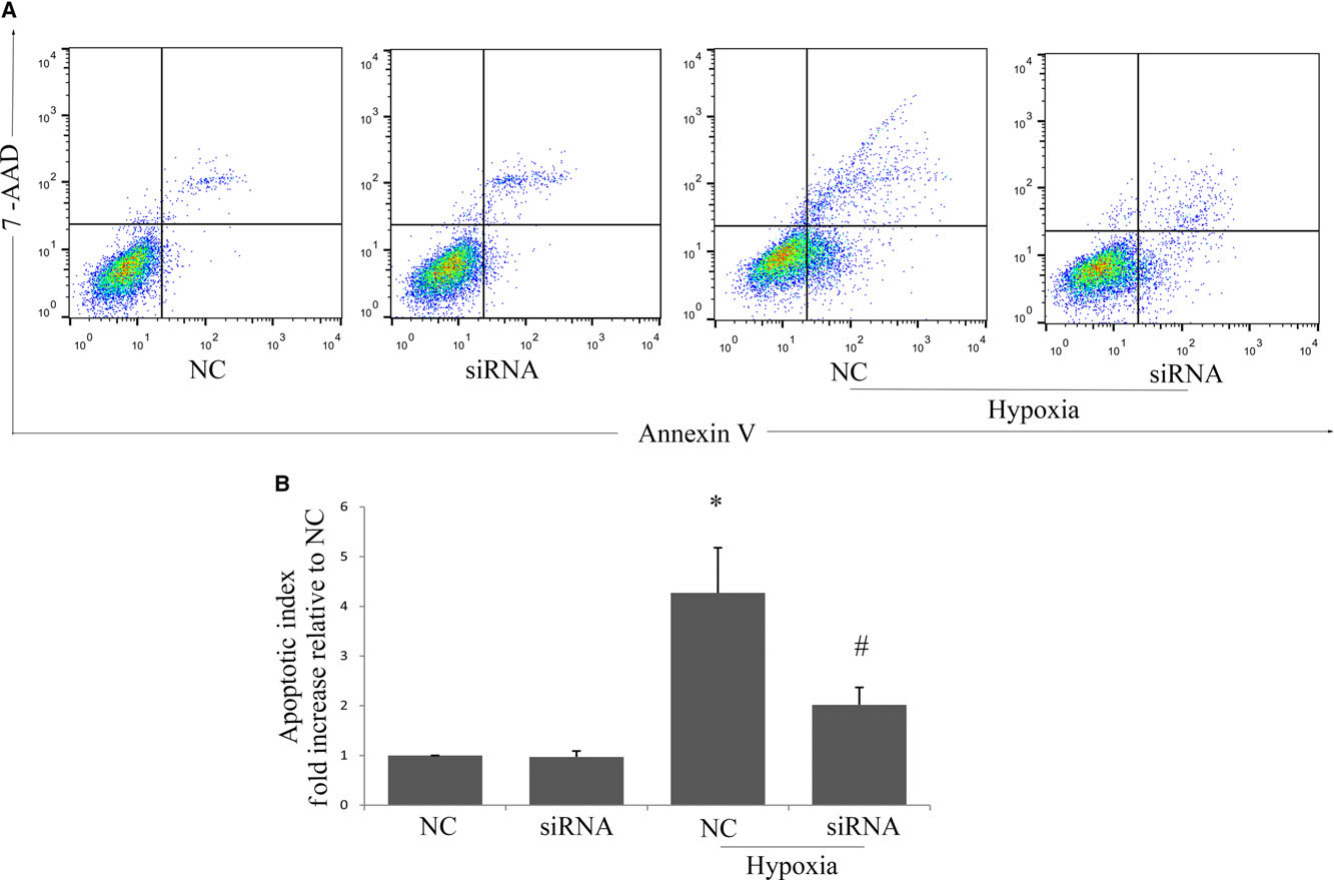
Effects of MR depletion on MSCs’ survival in vitro. (A) MSCs were exposed to hypoxia for 6 h after transfection with AAV‐ siRNA/GFP, cell injury was determined by flow cytometry after staining with Annexin V/7‐AAD. (B) Quantitative analysis of apoptotic cells by FACS analysis with Annexin V/7‐AAD staining. Data were collected from three independent experiments. *P < 0.05 vs NC, ^#^ *P* < 0.05 vs NC+hypoxia

To analyse the effect of MR on apoptosis of cultured MSCs, the MSCs transfected with AAV‐NC‐siRNA (NC group) or AAV‐MR‐siRNA (siRNA group) were treated with hypoxia in the absence of serum and glucose. Cell apoptosis was measured by FACS using the fluorescent dye PE Annexin V and 7‐AAD. The apoptotic cell number was significantly decreased in siRNA group compared with NC group in hypoxic condition (Figure 3A and B).

**Figure 4:**
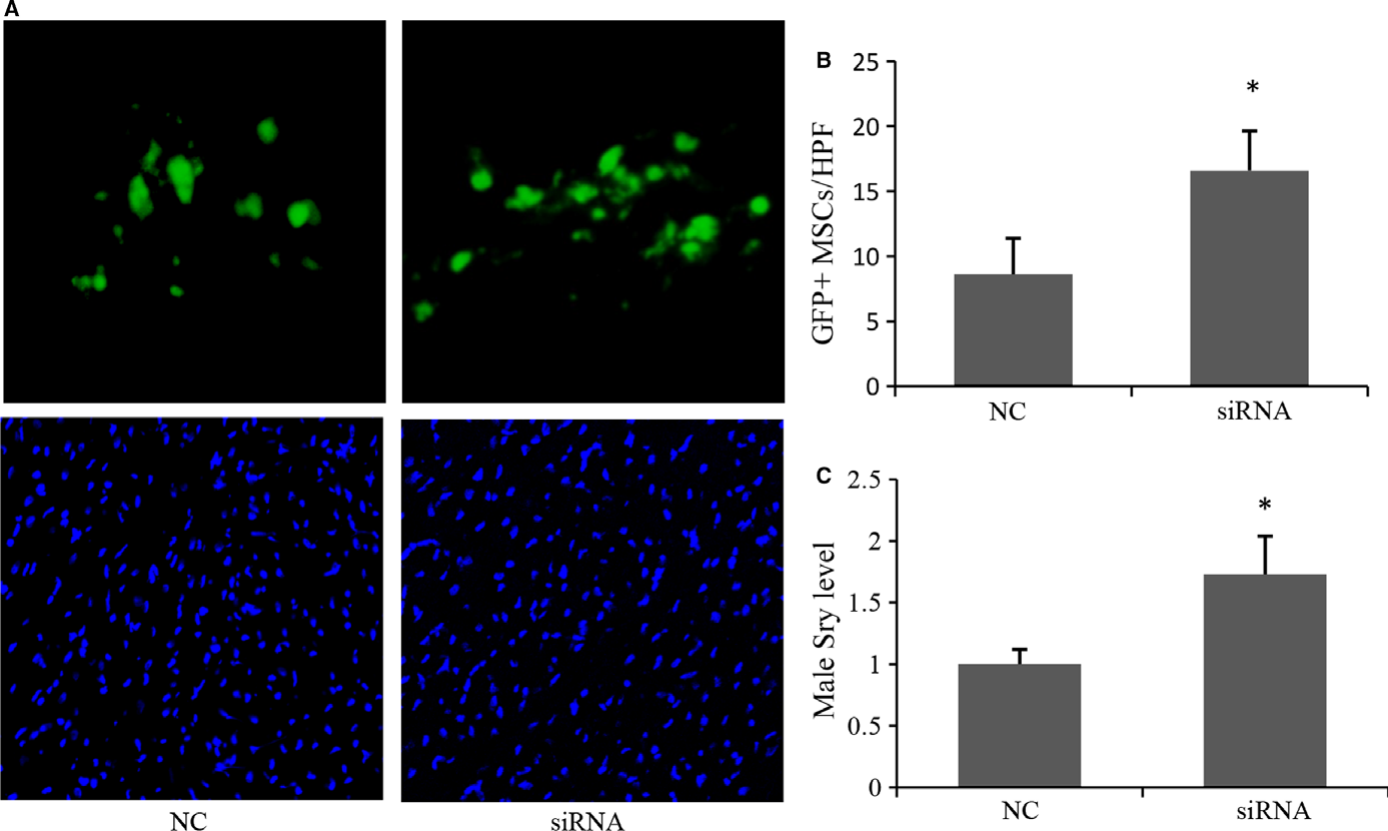
Effects of MR depletion on MSCs’ survival in vivo. (A) Representative images of GFP ^+^ MSCs in infarcted myocardium. (B) Quantitative analysis of MSCs survival 4 days after MI. (C) Real‐time PCR was used to detect the male Sry gene in female rat hearts 4 days after transplantation (n = 3). The β‐actin gene was used as internal control. *P < 0.05 vs NC

To further verify the cytoprotective effects of MR depletion on MSCs in vivo, MSCs from male rat hearts were injected into the female viable myocardium bordering infarction. Previous time‐course study showed that high level of graft cell death occurred for at least 4 days after grafting into injured hearts,^30^ and the number of survived stem cells at early time‐points directly correlated with subsequent LV functional improvement at later time‐points.^31^ Thus, in vivo donor cell survival was assessed 4 days after cell transplantation. It was observed that the number of GFP^+^ MSCs in the siRNA group was 1.92‐fold greater than that in NC group (Figure 4A and B). The in vivo MSCs survival was also determined by real‐time PCR for rat Sry gene. The results (Figure 4C) suggested that donor cell survival was higher in siRNA group as compared with NC group, with approximately 1.73‐fold enhancement of cell survival at 4 days after cell transplantation (*P* < 0.05).

### 3.3 Depletion of MR inhibited ROS generation and up‐regulated anti‐apoptotic proteins

**Figure 5:**
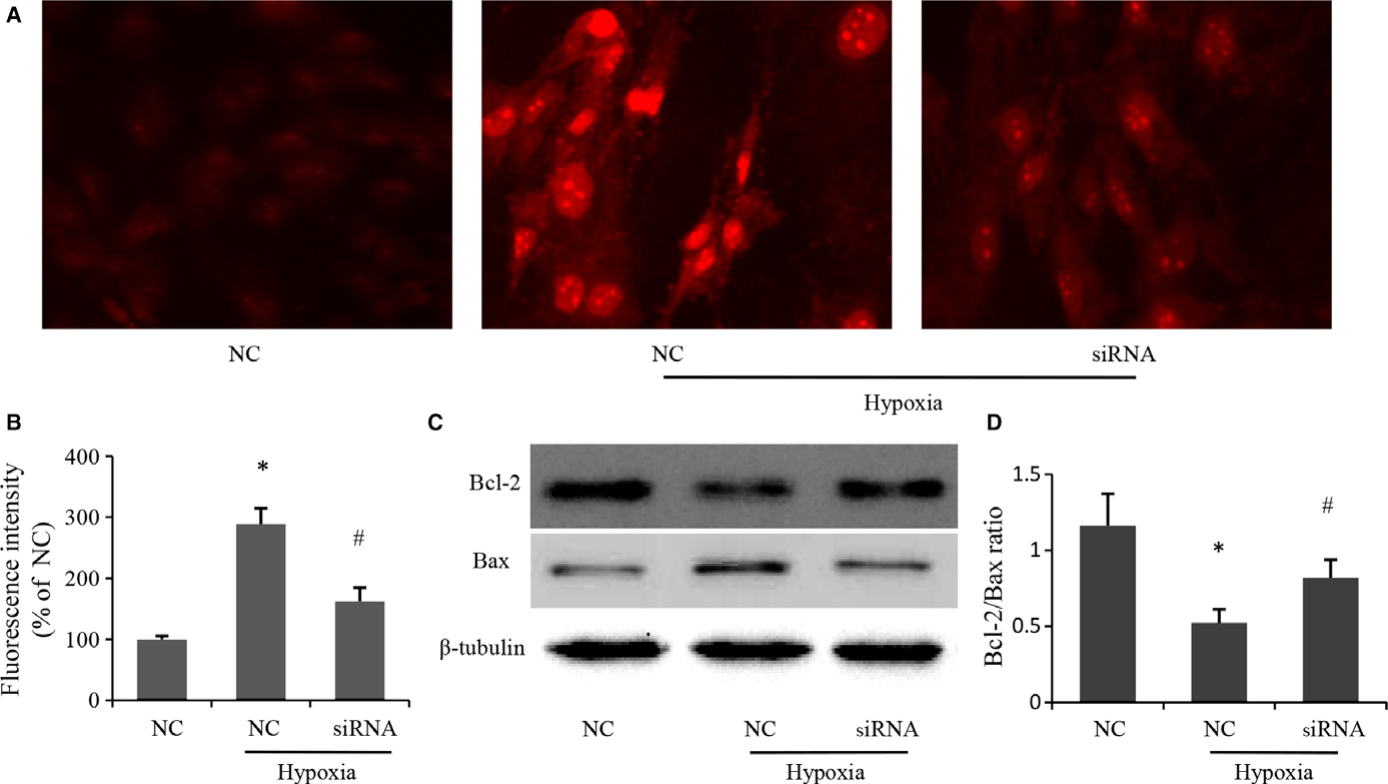
Effects of MR depletion on ROS production and expression of apoptotic proteins induced by hypoxia. (A) Representative photomicrographs of intracellular ROS in MSCs visualized by fluorescent microscopy. Red fluorescent intensity indicated ROS accumulation. (B) Quantitative analysis of red fluorescent intensity in individual group. Data were collected from three independent experiments. (C) Apoptotic proteins determined by immunoblotting in MSCs subjected to hypoxia or normoxia. (D) Quantitative analysis of Bcl‐2/Bax ratio using data from three independent experiments. *P < 0.05 vs NC, ^#^ *P* < 0.05 vs NC+hypoxia

Ischaemia could lead to ROS formation, which might intensify the anoikis signals in transplanted MSCs.^32^ So we examined whether MR silencing could influence the ROS formation in MSCs. As shown in Figure 5A and B, exposure of MSCs to hypoxia resulted in increased ROS generation, manifesting as cells with strong red fluorescent. Silencing MR restored intracellular ROS generation induced by hypoxia.

Furthermore, we examined the expression of apoptotic proteins. The expression of anti‐apoptotic protein Bcl‐2 was significantly decreased after hypoxia, which was markedly increased after MR depletion in hypoxic condition. Moreover, the increased level of Bax induced by hypoxia was attenuated when the MR expression was down‐regulated by siRNA (Figure 5C and D).

### 3.4 Depletion of MR improved the left ventricular function and reduced infarct size

**Figure 6:**
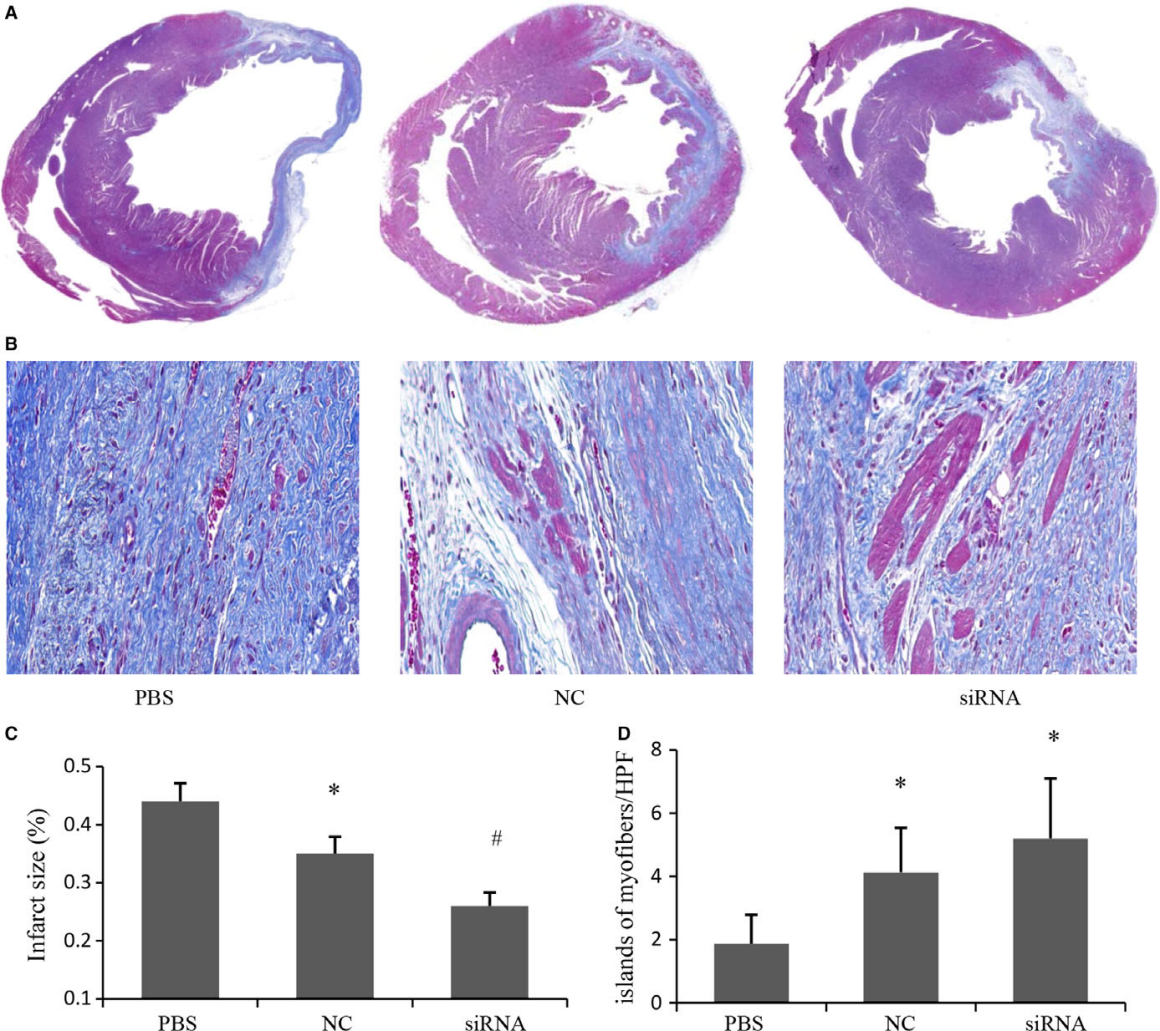
Effects of MR depletion on infarct size 4 weeks after cell transplantation. (A) Representative images of Masson staining from histological sections. (B) Representative images of myocyte islands in scar tissue from Masson staining histological sections. (C) Quantitative analysis of infarct size in various treatment groups (n = 5). (D) Quantitative analysis of myocyte islands in scar tissure (n = 5). *P < 0.05 vs PBS, ^#^ *P* < 0.05 vs NC

M‐mode echocardiography were examined 4 weeks after cell transplantation. LV function indices were summarized in Table 1. Both LVEF and LVFS were improved significantly in siRNA group and NC group when compared with PBS group, and the improvement of LVEF and LVFS was greater in siRNA group than that in NC group. LVID,s and LVID,d, markers of LV remodelling, were significantly decreased in siRNA group compared with PBS group and NC group though no statistic difference between PBS group and NC group. In addition, the infarct size was 23.49% lower in siRNA group than that in NC group, and both MSCs group showed significantly smaller infarct size (35.12 ± 2.96% in NC group; 26.87 ± 2.31% in siRNA group) compared with the PBS group (44.23 ± 3.17%) (Figure 6A and C). Besides, more surviving myocyte islands were observed after MSCs transplantation within the centre of infarcted tissue when compared with PBS group, and the amount of myocyte islands was even higher in siRNA group than that in NC group though it did not reach statistical significance (Figure 6B and D, *P* = 0.057).

**Table 1:** Echocardiographic findings in three experimental groups

	PBS	NC	siRNA
LVEF (%)	32.47 ± 1.91	37.56 ± 2.06*	44.81 ± 2.43#
LVFS (%)	15.13 ± 1.02	18.36 ± 0.61*	23.42 ± 1.24#
LVID,s	8.16 ± 0.74	7.96 ± 0.62	6.53 ± 0.73#
LVID,d	9.69 ± 0.84	9.61 ± 0.73	8.27 ± 0.79#

LVEF: left ventricular ejection fraction; LVFS: left ventricular fractional shortening; LVID,d: left ventricular internal diameter in diastole; LVID,s: left ventricular internal diameter in systole.

*P < 0.05 vs PBS, ^#^ *P* < 0.05 vs NC.

### 3.5 Depletion of MR promoted angiogenesis

**Figure 7:**
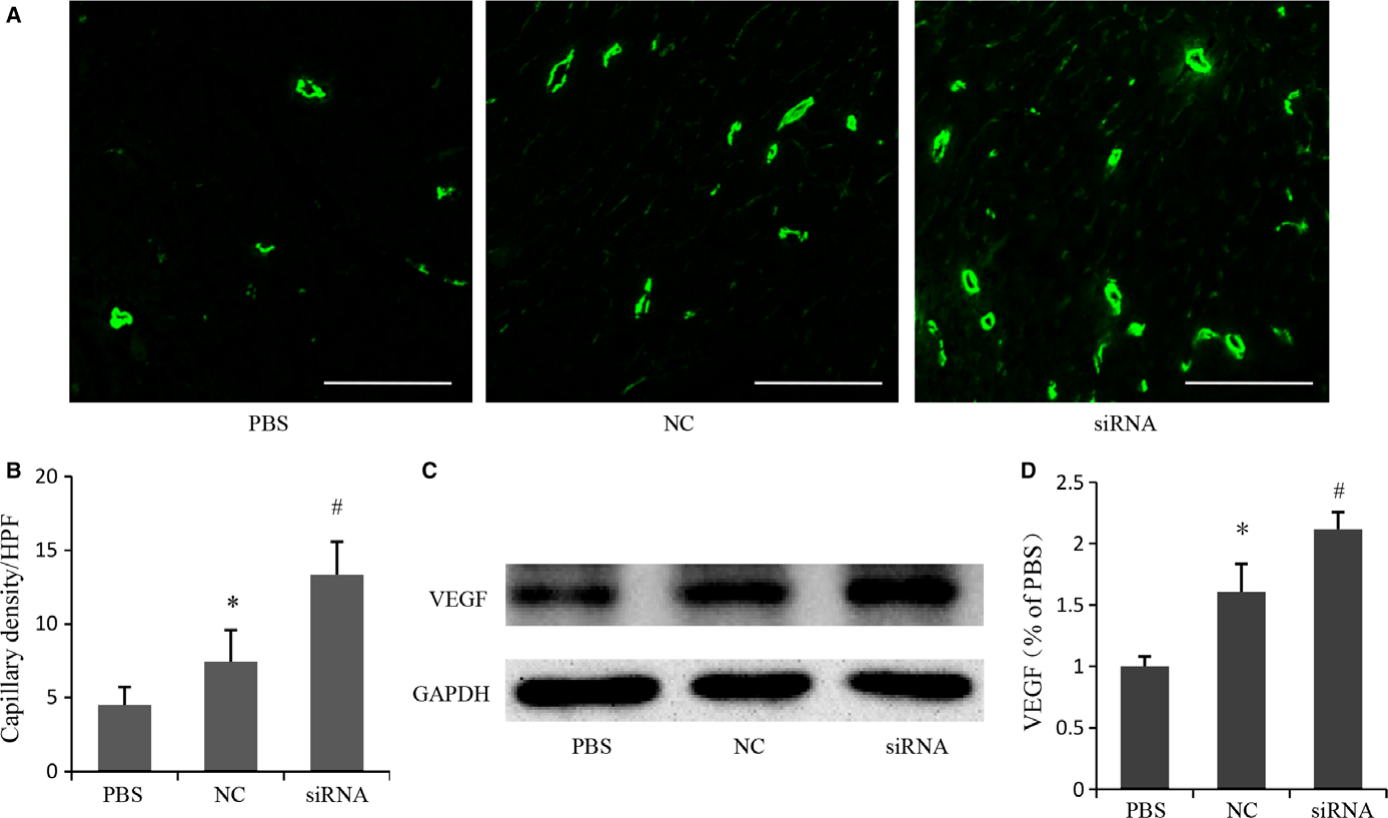
Effects of MR depletion on angiogenesis in the peri‐infarct area 4 weeks after cell transplantation. (A) Representative photomicrographs showing capillary density in different experimental groups based on CD31 immunostaining. Scale bar: 100 μm. (B) Quantitative analysis of the numbers of capillary vessels in various treatment groups (n = 5). (C) Representative electrophoresis graph of vascular endothelial growth factor by western blot. (D) Quantitative analysis for vascular endothelial growth factor (n = 5). *P < 0.05 vs PBS, ^#^ *P* < 0.05 vs NC

Capillary density was examined by immunostaining for CD31 in the peri‐infarcted areas 4 weeks after AMI. Representative images were shown in Figure 7A. Quantitative analyses revealed that capillary density was significantly higher in siRNA group (13.34 ± 2.23 vessels/HPF) and NC group (7.45 ± 2.13 vessels/HPF) when compared with PBS group (4.51 ± 1.21 vessels/HPF), and capillary density in siRNA group was even higher than that in NC group (Figure 7B). Accordingly, MSCs transplantation could up‐regulate VEGF expression in ischaemic myocardium at 4 weeks after AMI, which was significantly higher in siRNA group when compared with NC group (Figure 7C and D).

## 4 DISCUSSION

The efficacy of MSCs therapy were greatly associated with transplanted MSCs’ survival in local harsh microenvironment after AMI. Only few transplanted MSCs survived in damaged heart and mediated the cardioprotective effect.^27^, ^33^ Thus, promoting MSCs’ survival was more attractive than increasing MSCs quantity simply in order to improve the cardiac repair capacity of MSCs, considering the results of clinical trials which found a higher number of injected cells (up to 600 mol L^−1^) could potentially damage the cardiac function.^14^ Our study demonstrated that silencing the MR gene of MSCs could improve their survival under hypoxic environment, with a result of significant preservation of LV systolic function and attenuation of LV remodelling through angiogenesis in a rat AMI model. These data suggested that siRNA‐MR modified MSCs transplantation had great clinical value in improving the effectiveness of cell therapy in infarcted hearts. To our knowledge, the present study firstly examined the potential benefits of silencing MR for MSCs‐based therapy in ischaemic heart disease.

Myocardial infarction resulted in a hypoxic area containing a central focus of near‐zero O_2_ pressure bordered by tissue with diminished but nonzero O_2_ pressures.^34^ So, the donor cells injected into border zones was under an hypoxic rather than anaerobic environment with deficiency of nutrient substance. Our in vitro model of MSCs injury using a sealed, hypoxic Anaerobic Rectacular Jar could partly mimic the hypoxic environment that cells might experience in the injured hearts, and the preliminary experiments also proved that hypoxia plus serum/glucose deprivation was a reproducible method to induce MSCs injury in vitro.

Besides an oxidative stress status in ischaemic environment with deficiency of oxygen and nutrient substance, masses of proapoptotic or cytotoxic factors in the infarcted myocardium could also induce donor cell death.^35^ Furthermore, the present study revealed that MSCs expressed MR as demonstrated by qRT‐PCR, and MR activation was also involved in the process of hypoxia‐induced MSCs apoptosis. Furthermore, MR knock‐down by siRNA could strengthen the ability of MSCs to resist hypoxia and enhance the transplanted MSCs’ survival in damaged myocardium. As a result, more stem cells exerted their trophic supports for infarcted hearts, as confirmed by increased VEGF expression, which was important for the functional improvement of infarcted heart. Besides promoting the formation of collateral vessels, VEGF also provided cytoprotective effects on MSCs and cardiomyocytes.^36^, ^37^ These all led to a decreased infarct size and preserved cardiac function.

Oxidative stress was one of the important pro‐apoptotic factors in ischaemic conditions. Oxidative stress resulted in excessive production of ROS, which could damage the inner mitochondrial membrane potential and induce MSCs apoptosis.^32^ MR activation was proven to mediate the ROS production in many pathological conditions,^38^, ^39^, ^40^ Consistent with the previous reports, the present study showed hypoxia led to the accumulation of ROS in MSCs, MR silencing could decrease the ROS production and inhibit hypoxia‐induced apoptosis. As known, Bcl‐2 and Bax are two members of the Bcl‐2 family, and the Bcl‐2/Bax ratio was important in determining susceptibility to apoptosis. Our results suggested that MR depletion led to an increase in Bcl‐2/Bax ratio in hypoxic condition, which could avoid cell apoptosis. Taken together, these data indicated that the protective effects of MR depletion were associated with decreased oxidative stress reaction and increased ratio of Bcl‐2/Bax.

Conflicting data about the influence of MR activation on stem cells had been reported. MR antagonists such as eplerenone or spironolactone could increase the number and improve the function of EPCs.^21^, ^41^ Instead, MR activation could also improve migration and modify the secretory activity of cardiac progenitor cells.^42^, ^43^ The discrepancy might be because of different stem cell types and study design (in vitro vs in vivo). Furthermore, short‐term as opposed to chronic exposure to MR activation might also explain the inconsistency among studies. For instance, in previous studies, short‐term exposure to aldosterone most often led to vasodilation mediated by endothelium‐derived nitric oxide, while chronic exposure to aldosterone impaired endothelial functions.^44^, ^45^

In summary, MR activation was involved in MSCs’ death after transplantation, MR depletion could promote MSCs’ survival by alleviating hypoxia‐induced oxidative stress and increasing Bcl‐2/Bax ratio. As a treatment, transplantation of siRNA‐MR modified MSCs was associated with improvement of LV function and reduced infarct size. Thus, our study provided evidence that MR might be a potential target for enhancing the efficacy of cell therapy in ischaemic diseases.

## CONFLICTS OF INTEREST

The authors confirm that there are no conflicts of interest.

## AUTHOR CONTRIBUTIONS

Xinxing Xie, Yunli Shen, and Zheyong Huang conceived and designed the study. Xinxing Xie, Yunli Shen, and Jing Chen performed the experiments. Xinxing Xie, Yunli Shen, and Zheyong Huang analysed the results. Xinxing Xie and Yunli Shen wrote the manuscript. Junbo Ge supervised the experiment.
